# Exploring research dynamics of PDT in anti-infective applications (2004–2024): a bibliometric perspective

**DOI:** 10.3389/fphar.2025.1510690

**Published:** 2025-06-20

**Authors:** Wen Wen, Shuang Liang, Yun Zhang, Meiyuan Lin, Yuan Gao, Jun Deng

**Affiliations:** Department of Respiratory and Critical Care Medicine, The Affiliated Hospital of Southwest Medical University, Luzhou, Sichuan, China

**Keywords:** photodynamic therapy, infections, antibacterial, photothermal therapy, bacteria resistance, biofilm, nanoparticles

## Abstract

**Background:**

Photodynamic therapy (PDT) has become a popular research direction in the field of infection control; however, there is still a lack of systematic bibliometric analysis. This paper aims to fill this gap by conducting the first comprehensive bibliometric analysis of studies related to PDT in infection control over the past 20 years, in order to gain in-depth insights into its current status and emerging trends.

**Methods:**

Publications related to PDT and infection control were collected from the Web of Science Core Collection database (WoSCC) from January 2004 to April 2024. Microsoft Excel was used to organize the data and analyze annual publication trends, while VOSviewer and CiteSpace were employed for the visual analysis of the selected literature.

**Results:**

A total of 9,711 articles and reviews meeting the inclusion criteria were retrieved for this study. Over the past two decades, publications in the field of PDT for infection control have exhibited a marked upward trajectory. China leads globally in research output and influence within this domain, establishing robust collaborative networks with the United States and Brazil. The most productive institution, journal, and author were the Universidade de São Paulo, *Photodiagnosis and Photodynamic Therapy*, and Michael R. Hamblin, respectively. Early studies frequently featured keywords such as “5-aminolevulinic acid,” “photosensitization,” “toluidine blue O,” and “photodynamic inactivation.” In contrast, recent research has shifted toward innovative themes such as “nanoparticles,” “antibacterial nanomaterials,” “aggregation-induced emission,” and “photothermal therapy.” Nanotechnology-based synergistic enhancement strategies for PDT and antimicrobial photodynamic therapy (aPDT) represent a current research hotspot. Nine emerging themes — “aggregation-induced emission,” “wound healing,” “photothermal therapy,” “sonodynamic therapy,” “antioxidant,” “formulation,” “design,” “nanosheets,” and “graphene oxide”—have notably influenced future directions and warrant special attention.

**Conclusion:**

This study is the first to comprehensively summarize the research trends and progress in PDT for anti-infective treatment through bibliometric analysis, and to clarify recent research frontiers and hot directions, providing a valuable reference for the continued exploration of PDT applications in anti-infective therapy.

## 1 Introduction

Infectious diseases remain one of the major global challenges to human health. Although conventional antimicrobial therapies, particularly antibiotics, have significantly reduced infection-related mortality in recent decades, the spread of drug-resistant pathogens has increased markedly due to antibiotic overuse and rapid microbial evolution through genetic mutations and horizontal gene transfer (Friedman et al., 2016; Ferri et al., 2017; Alanis, 2005). The emergence of multidrug-resistant (MDR) bacteria has severely compromised the efficacy of traditional antibiotics (Tängdén et al., 2024; Murray et al., 2022). Notably, highly resistant strains such as methicillin-resistant *Staphylococcus aureus* (MRSA) and vancomycin-resistant *Enterococcus* (VRE) pose substantial clinical challenges, establishing them as priority targets for global public health prevention and control efforts (Guo et al., 2020; Chambers and Deleo, 2009; Ahmed and Baptiste, 2018). Antimicrobial resistance (AMR) is currently responsible for approximately 700,000 deaths annually worldwide. A government-commissioned report in the United Kingdom projected that, without effective intervention strategies, AMR-related fatalities could rise to 10 million per year by 2050 (Willyard, 2017; O’Neill, 2014). Although these estimates remain debated (de Kraker et al., 2016), AMR undeniably represents a grave public health threat. This threat disproportionately affects elderly and immunocompromised populations, manifesting primarily through elevated rates of treatment failure and complications (Duhaniuc et al., 2024; World Health Organization, 2023; Nooruzzaman et al., 2024). Consequently, there is an urgent need to develop new antimicrobial alternatives. However, the protracted timeline and high costs associated with new antibiotic development have caused therapies to lag behind the rapid evolution of resistance, exacerbating the global AMR crisis (Tängdén et al., 2024). Furthermore, the growing prevalence of biofilm-associated infections presents additional clinical challenges. Pathogenic bacteria can form structurally complex biofilms within host tissues, conferring enhanced resistance to both antibiotic therapy and host immune defenses, ultimately leading to persistent infections and frequent recurrences (Nesse et al., 2023; Cieplik et al., 2018; Mah and O'Toole, 2001). Conventional antibiotic regimens often exhibit poor clinical efficacy against such infections, highlighting the limitations of traditional antimicrobial approaches in managing complex cases (Rabin et al., 2015; Uruén et al., 2020; Lewis, 2001). Alarmingly, the frequent coexistence of AMR and biofilm formation further compounds these challenges. Confronted with the dual threats of multidrug resistance and biofilm-associated infections, the development of novel treatment strategies has become an urgent priority.

Photodynamic therapy (PDT) is a non-antibiotic treatment that employs photosensitizers (PSs) to interact with specific wavelengths of light, generating reactive oxygen species (ROS) in the presence of oxygen, which effectively eliminate pathogens (Cieplik et al., 2018; Wainwright and Crossley, 2004). Its antibacterial mechanism primarily operates through Type I (electron transfer) and Type II (energy transfer) photochemical reactions (Wainwright, 1998; Cieplik et al., 2018). Initially, PDT was predominantly used for cancer treatment, and its antimicrobial effects were largely overlooked. However, in the 1990s, PDT garnered renewed interest due to its significant success in treating drug-resistant pathogens (Wainwright et al., 2017) Compared to traditional antibiotics, PDT offers substantial advantages in combating antibiotic-resistant pathogens and biofilm-associated infections (Zanin et al., 2005; Cieplik et al., 2014). Notably, due to its unique antimicrobial mechanism, no microbial resistance has been observed to date (Maisch, 2015). It has been successfully applied to treat various infections, demonstrating notable efficacy in wound and burn infections (Hamblin et al., 2003), oral infections (Javed et al., 2014) (such as periodontitis and gingivitis), skin infections (Maisch et al., 2004), and persistent biofilm infections (Bair et al., 2020) that are difficult to treat with antibiotics. Despite these advancements, PDT faces several challenges in practical applications, including the selection of PSs and non-specific targeting, optimization of light conditions, and limitations in the lifetime and range of action of singlet oxygen (^1^O_2_). With advancements in nanotechnology and light source technologies, the efficacy of antimicrobial PDT (aPDT) has been notably improved (Liu et al., 2024; An et al., 2024; Chen et al., 2024). In recent years, research in this area has shown rapid growth. The increasing research activity highlights its potential and broad application prospects in antimicrobial treatments.

Bibliometric analysis is a quantitative research tool used to examine scientific literature and academic communication, aiming to reveal research trends, hotspots, and developmental directions within specific fields (Li et al., 2020). By analyzing information such as citations, authors, journals, institutions, and keywords, researchers can assess disciplinary dynamics from a global perspective, identify high-impact research outputs and scholars, and explore the formation and evolution of academic collaboration networks. In recent years, while bibliometric analyses have been conducted on the application of PDT in diseases such as skin cancer, periodontitis, lung cancer, and cervical cancer (Sun et al., 2023; Shanazarov et al., 2024; Gu et al., 2024), there remains a gap in bibliometric analysis regarding PDT in the field of anti-infection. Therefore, this study, for the first time, employs bibliometric analysis based on the Web of Science Core Collection (WoSCC) database to evaluate the research impact and innovation of PDT in anti-infective treatment from multiple perspectives, including countries, institutions, authors, journals, and keywords. It provides a comprehensive overview of the research status in this field, reveals current research hotspots, and predicts future research directions, offering important references and in-depth insights for subsequent research.

## 2 Materials and methods

### 2.1 Data sources and retrieval strategies

The WoSCC is a comprehensive, multidisciplinary database that offers extensive publication information, including detailed references to authors, institutions, journals, and publishers. Its powerful features and citation analysis tools enable researchers to efficiently identify high-impact studies, track the research focus of leading scholars globally, and monitor emerging trends in their fields. As a widely recognized data source for bibliometric analysis, WoSCC has been extensively utilized in academic research.

**FIGURE 1:**
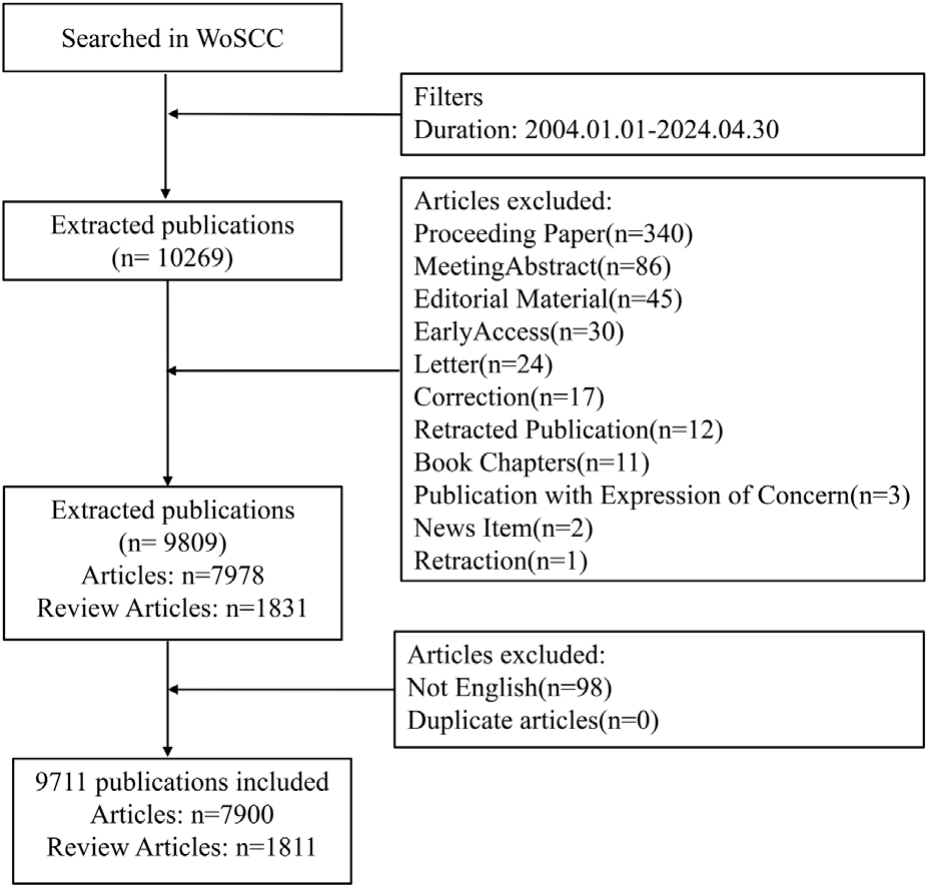
A flowchart illustrating the search strategies utilized in the Web of Science Core Collection (WoSCC) database. WoSCC = Web of Science Core Collection.

To minimize information bias due to database updates, data retrieval was completed in the WoSCC on 19 March 2025. The specific search strategy was as follows: TS=((“pathogen*” OR “bacteria*” OR “bacterial infections” OR “bacterial tolerance” OR “infectious diseases” OR “infection*” OR “antibacterial” OR “anti-bacterial” OR “antimicrobial” OR “anti-infective” OR “antimicrobial activity” OR “antibacterial activity” OR “antibiotic resistance” OR “antimicrobial resistance” OR “fungi” OR “fungal infections” OR “antifungal resistance” OR “virus*” OR “viral infections” OR “parasite*”) AND (“photodynamic therapy” OR “PDT” OR “antimicrobial photodynamic therapy” OR “aPDT” OR “photodynamic antimicrobial chemotherapy” OR “PACT” OR “photodynamic inactivation” OR “PDI”)), Time range = January 2004 to April 2024, Document Type = (Article OR Review), and Language = English. Detailed information regarding literature screening is presented in Figure 1. All screening procedures were independently conducted by two investigators, with any disagreements resolved through discussion or, if necessary, arbitration by a third investigator.

### 2.2 Data extraction and collection

Two researchers exported the “full-text records and cited references” of the target publications (n = 9,711 articles) in plain text format for subsequent analysis. The impact factor (IF) and subject category quartile rankings of the journals were obtained from the latest edition of the Journal Citation Reports (JCR). The H-index, calculated based on citation data from Web of Science, refers to the number of articles (h) published by a researcher, each of which has been cited at least h times. It is a key indicator for quantifying the productivity and impact of scholarly output by a researcher or academic journal.

### 2.3 Data analysis and visualization

All data were imported into Microsoft Excel, VOSviewer, and CiteSpace for analysis. Excel was used to categorize and manage target indicators, including countries/regions, institutions, authors, journals, keywords, and references, as well as to chart annual publications. Utilizing VOSviewer (version 1.6.18), network, density, and overlay visualization maps were constructed by analyzing co-authorship networks among countries, institutions, and journals, co-citations of journals and authors, and co-occurrence relationships of keywords. CiteSpace (version 6.4 R1) was applied to detect keyword bursts and identify research frontiers and emerging trends in the field of PDT for anti-infection treatment. In the visual map constructed by VOSviewer, colors distinguish clusters, circles represent nodes, the size of the circles indicates the frequency of occurrences, and the thickness of the connecting lines reflects the strength of the relationships between the nodes.

## 3 Results

### 3.1 Annual growth trend

**FIGURE 2:**
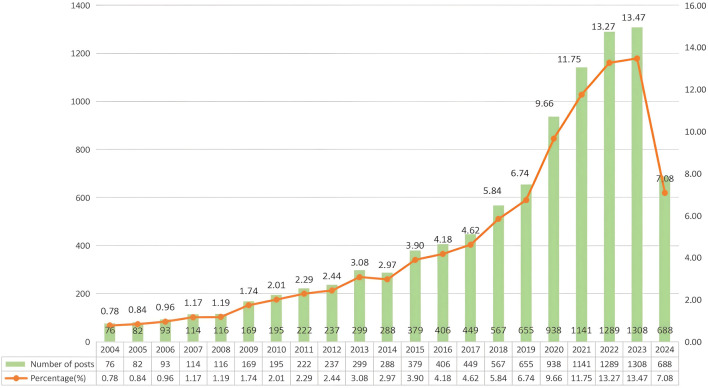
Annual publication volume of PDT in infection control applications from January 2004 to April 2024.

A total of 9,711 English-language publications on PDT for anti-infective applications were included in this study, comprising 7,900 original articles (81.35%) and 1,811 review articles (18.65%). Figure 2 illustrates the annual distribution and cumulative publication count for studies on PDT in anti-infective research. Over the past two decades, research output in this field has demonstrated a significant upward trend. Specifically, from 2004 to 2013, annual publications increased steadily, followed by a minor decline in 2014. From 2015 to 2020, publication numbers rebounded noticeably, accompanied by an accelerating rate of growth. Between 2021 and 2023, annual publication output remained at elevated levels, exceeding 1,000 publications each year and peaking at 1,308 in 2023. Although data from the first 4 months of 2024 are insufficient for evaluating full-year trends, current publication rates already exceed 50% of 2023’s total output, indicating that research activity remains robust. Overall, PDT anti-infection research exhibits a consistent year-over-year increase, underscoring the field’s growing significance and sustained scholarly engagement.

### 3.2 Analysis of countries/regions

**FIGURE 3:**
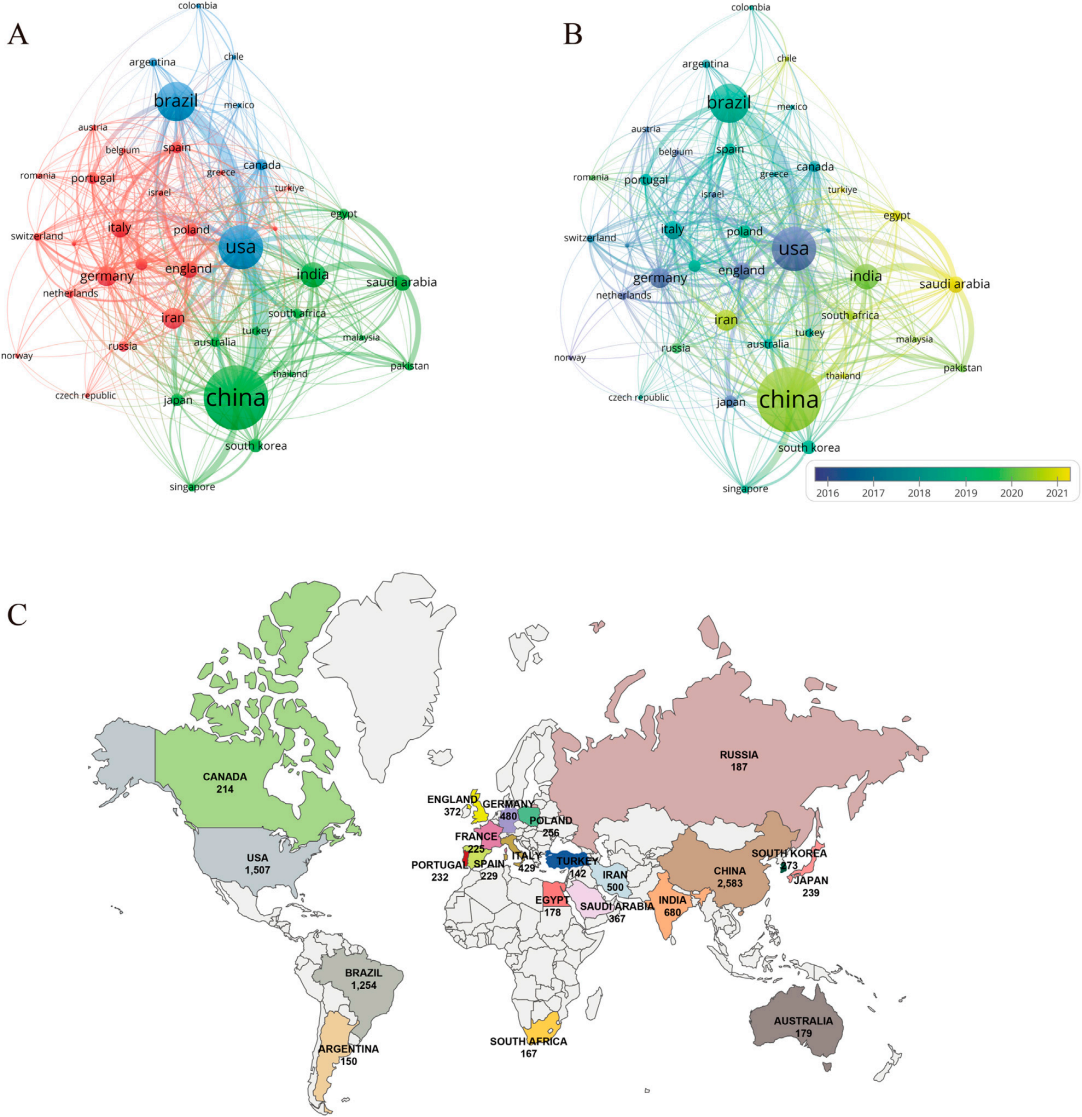
Analysis of countries/regions and institutions. **(A)** Cooperation network diagram among countries/regions with no fewer than 40 articles (n = 41); **(B)** the temporal map of collaboration network among countries/regions with no less than 40 articles (n = 41); **(C)** Distribution map of research forces in various countries with no less than 150 published papers in the field of PDT anti-infection.

A total of 126 countries have participated in research on PDT for anti-infective therapy. Table 1 presents the top 10 countries in terms of research output. China ranks first in both the number of publications (n = 2,583) and total citations (98,331), followed by the United States (n = 1,507, 82,079) and Brazil (n = 1,254, 31,463). The combined publication output of these three countries accounts for over 50% of the total publications, highlighting their significant involvement and leadership in this research field. In terms of average citations per article, the United States (54.47), England (48.56), and Germany (46.29) rank in the top three, indicating that their research began earlier and their results are relatively well-developed. Regarding the H-index, which reflects both productivity and academic impact, the top five countries are China (H = 131), the United States (H = 129), Germany (H = 76), Brazil (H = 74), and England (H = 68). Figure 3A shows the co-authorship network among countries with no fewer than 40 publications. Network analysis reveals strong collaborations between China, the United States, and Brazil, consistent with their high productivity. The historical trend (Figure 3B) indicates that the early contributions to this field primarily came from developed countries such as the United States, England, Germany, and Japan. Although China entered this field relatively late, it has experienced rapid development in recent years, with its research output and academic influence now ranking among the leaders internationally. In recent years, countries such as Saudi Arabia and Egypt have also emerged as new contributors to international research collaborations. Figure 3C shows the distribution of countries with more than 150 publications, providing a clear reference for analyzing global research strength.

**TABLE 1:** The top 10 countries and regions in terms of the number of publications.

Rank	Country	Publications	Percentage (n = 9,711)	Total citations	Mean citations	H-index	Total link strength
1	China	2,583	26.60%	98,331	38.07	131	776
2	United States	1,507	15.52%	82,079	54.47	129	1,121
3	Brazil	1,254	12.91%	31,463	25.09	74	444
4	India	680	7.00%	19,256	28.32	65	365
5	Iran	500	5.15%	13,002	26.00	49	196
6	Germany	480	4.94%	22,217	46.29	76	347
7	Italy	429	4.42%	14,613	34.06	57	318
8	England	372	3.83%	18,064	48.56	68	383
9	Saudi Arabia	367	3.78%	6,741	18.37	40	338
10	South korea	273	2.81%	9,782	35.83	48	149

### 3.3 Analysis of institutions

**FIGURE 4:**
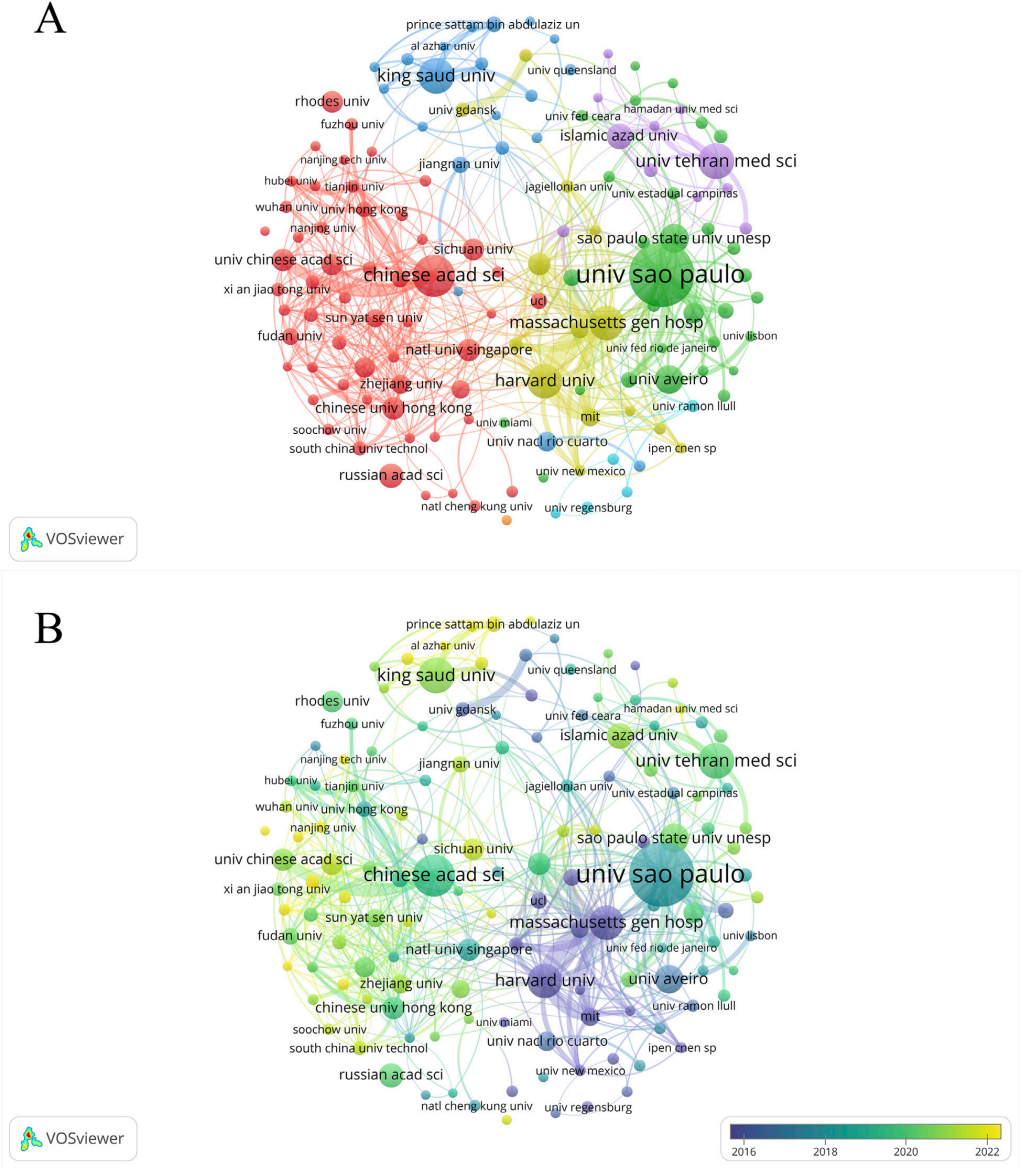
Analysis of institutions. **(A)** Cooperation network diagram among institutions with no fewer than 25 articles (n = 136); **(B)** the temporal map of collaboration network among institutions with no less than 25 articles (n = 136).

A total of 7,141 institutions have engaged in research on PDT for anti-infective therapy. As shown in Table 2, the top 10 institutions in terms of publication output exhibit a multipolar distribution: the United States, Brazil, and Iran each hold two positions. One institution each from China, Saudi Arabia, Portugal, and Russia is also included. Among these, the Universidade de São Paulo ranks first globally with 433 publications (4.46%), followed by the Chinese Academy of Sciences (228, 2.35%), King Saud University (181, 1.86%), and Tehran University of Medical Sciences (180, 1.85%). Notably, although Massachusetts General Hospital (169, 1.74%) and Harvard University (168, 1.73%) in the United States rank fifth and sixth in terms of publication output, their research impact indicators are outstanding: the total citation counts are 19,850 and 19,231, respectively, with average citations per article reaching 117.46 and 114.47, and H-index of 83 and 88. These three core metrics consistently place them among the top two positions globally, highlighting the significant academic standing of the United States institutions in this research field. To further investigate inter-institutional collaboration, a co-authorship analysis was conducted on all publications. Figure 4A shows that 136 institutions have published at least 25 papers. These 136 institutions form 7 clusters, with the red cluster being the largest, consisting of 55 institutions, primarily from China. The United States institutions exhibit the most intense collaboration (yellow cluster). The analysis of historical contributions (Figure 4B) reveals that several United States research institutions, led by Massachusetts General Hospital, have dominated the early development of PDT anti-infective research. Meanwhile, research forces from Saudi Arabia and China began to participate more actively in this field starting in 2022.

**TABLE 2:** The top 10 institutions ranked by the number of publications.

Rank	Institutions	Location	Publications	Percentage (n = 9,711)	Total citations	Mean citations	H-index	Total link strength
1	Universidade de Sao Paulo	Brazil	433	4.46%	12,534	28.95	56	323
2	Chinese Academy of Sciences	China	228	2.35%	13,653	59.88	69	195
3	King Saud University	Saudi Arabia	181	1.86%	4,190	23.15	35	91
4	Tehran University of Medical Sciences	Iran	180	1.85%	3,373	18.74	34	115
5	Massachusetts General Hospital	United States	169	1.74%	19,850	117.46	83	477
6	Harvard university	United States	168	1.73%	19,231	114.47	88	354
7	Universidade Estadual Paulista	Brazil	132	1.36%	2,483	18.81	48	80
8	Universidade de Aveiro	Portugal	129	1.33%	6,297	48.81	43	55
9	Islamic Azad University	Iran	110	1.13%	2,319	21.08	26	65
10	Russian Academy Of Sciences	Russia	99	1.02%	2,389	24.13	24	3

### 3.4 Analysis of journals

**FIGURE 5:**
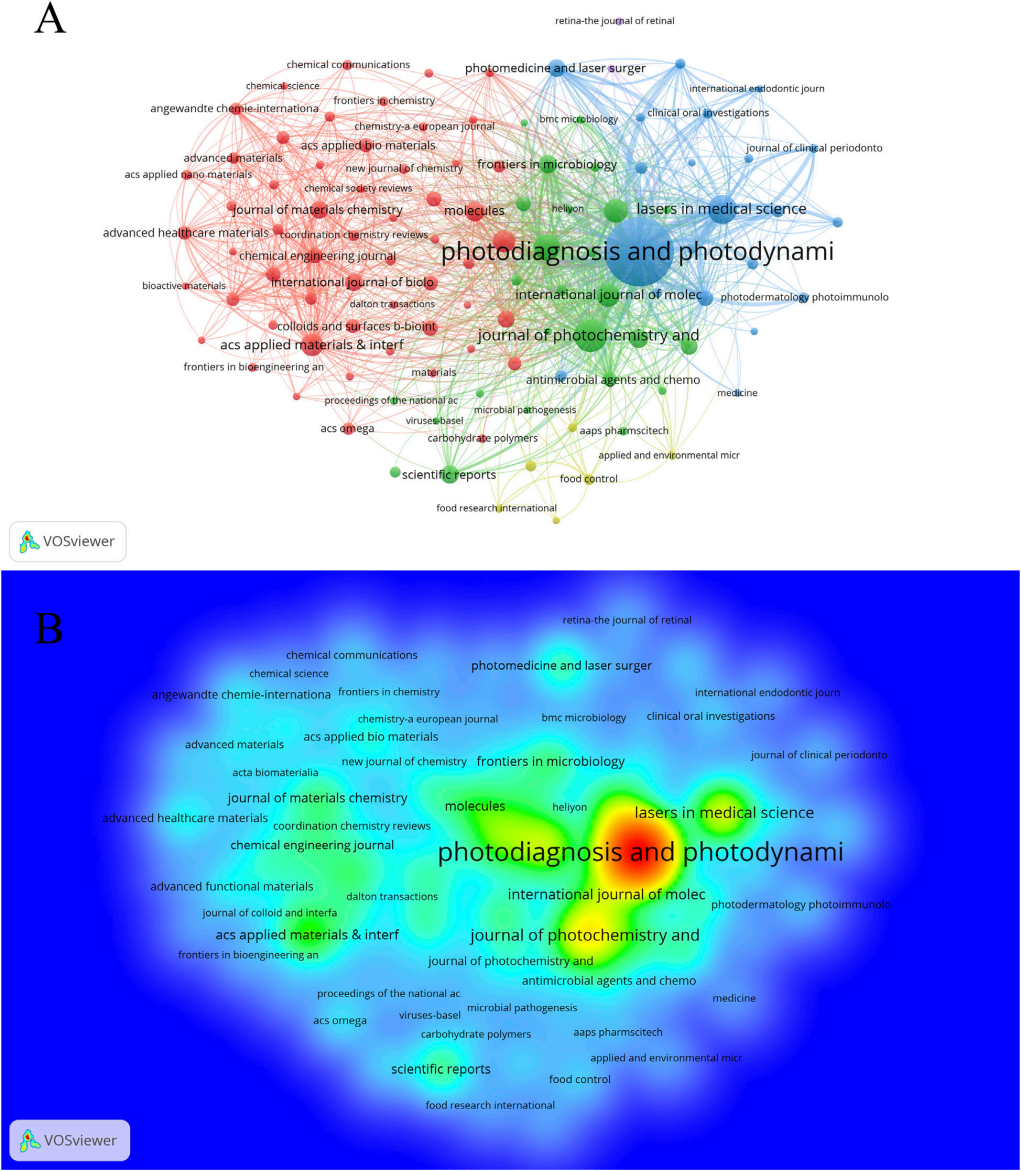
Analysis of journals. **(A)** Cooperation network diagram among journals with no fewer than 15 articles (n = 111); **(B)** The density visualization map (n = 111).

**FIGURE 6:**
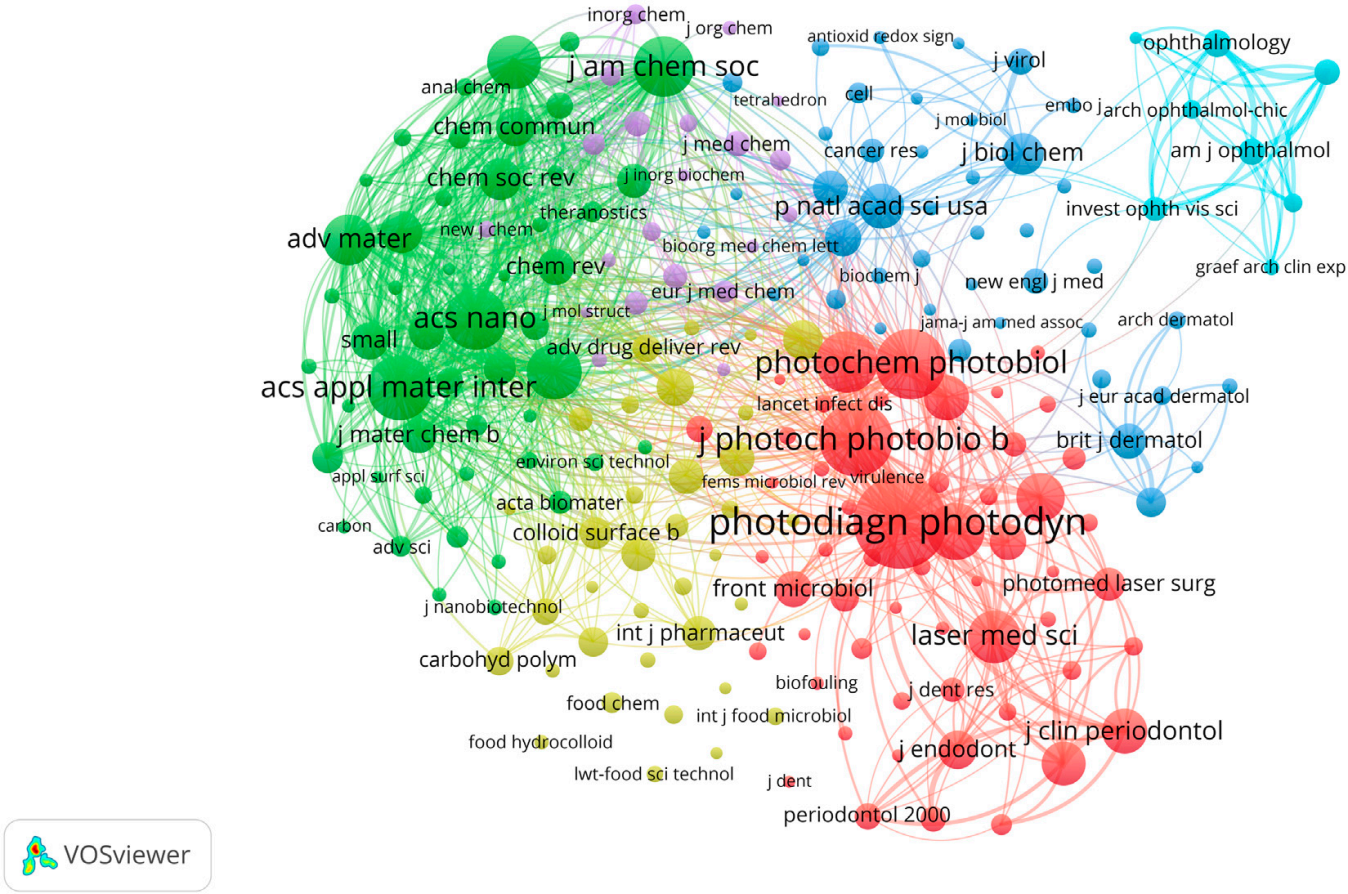
Analysis of journals. Co-citation analysis of journals with over 500 co-citations (n = 215).

A total of 1,903 journals have contributed to the research on PDT in the field of anti-infection treatment. The top 10 journals with the highest publication volumes in this field are presented in Table 3. *Photodiagnosis and Photodynamic Therapy* leads the field in antimicrobial photodynamic research with 998 publications, 18,089 citations, and an H-index of 49 — all core metrics ranking first among peer journals. The *Journal of Photochemistry and Photobiology B: Biology* ranks second, with 234 publications and an H-index equal to that of the leading journal (H = 49). *Lasers in Medical Science* ranks third, with 191 publications and an H-index of 41. Notably, *Photochemical and Photobiological Sciences* ranks fourth with 158 publications, but it has the highest average citations per article and the second-highest TLS, highlighting its “high quality with fewer publications” characteristic. Among the Top 10 high-producing journals, five journals are ranked in Quartile 1 (Q1) of the JCR. The IF of these 10 journals ranges between 2.1 and 8.5, with *ACS Applied Materials and Interfaces* becoming the benchmark journal for academic influence in this group with an IF of 8.5. Furthermore, there are 111 journals in this field with more than 15 publications. A journal collaboration network (Figure 5A) and density visualization map (Figure 5B) were constructed using VOSviewer. In the density map, red areas indicate higher co-occurrence frequencies, meaning the journal is cited or mentioned more frequently. *Photodiagnosis and Photodynamic Therapy* occupies a dominant position in the visualization map, fully demonstrating its central role and strong academic influence. Co-citation analysis (Figure 6) further reveals that 215 journals form a closely connected academic community. The top five co-cited journals were *Photodiagnosis and Photodynamic Therapy, Journal of Photochemistry and Photobiology B: Biology, Photochemistry and Photobiology, ACS Applied Materials and Interfaces, and ACS Nano.*

**TABLE 3:** The top 10 journals in terms of the number of publications.

Rank	Journals	Location	Publications	Percentage (n = 9,711)	Total citations	Mean citations	IF (2023)	JCR	H-index	Total link strength
1	*Photodiagnosis and Photodynamic Therapy*	Netherlands	998	10.28%	18,089	18.13	3.1	Q2	49	9,574
2	*Journal of Photochemistry and Photobiology B: Biology*	Switzerland	234	2.41%	7,614	32.54	3.9	Q1/Q2	49	3,929
3	*Lasers in Medical Science*	England	191	1.97%	5,429	28.42	2.1	Q2/Q3	41	3,280
4	*Photochemical and Photobiological Sciences*	England	158	1.63%	8,090	51.20	2.7	Q2/Q3	45	4,042
5	*Photochemistry and photobiology*	United States	128	1.32%	3,706	28.95	2.6	Q3	36	2,268
6	*International journal of Molecular sciences*	Switzerland	125	1.29%	3,847	30.78	4.9	Q1/Q2	32	1,681
7	*ACS Applied Materials and Interfaces*	United States	117	1.20%	5,191	44.37	8.5	Q1	45	1,375
8	*Pharmaceutics*	Switzerland	116	1.19%	2,564	22.10	4.9	Q1	24	1,219
9	*Molecules*	Switzerland	90	0.93%	3,736	41.51	4.2	Q2	27	1,107
10	*Plos One*	United States	84	0.86%	2,431	28.94	2.9	Q1	31	878

### 3.5 Analysis of authors

**FIGURE 7:**
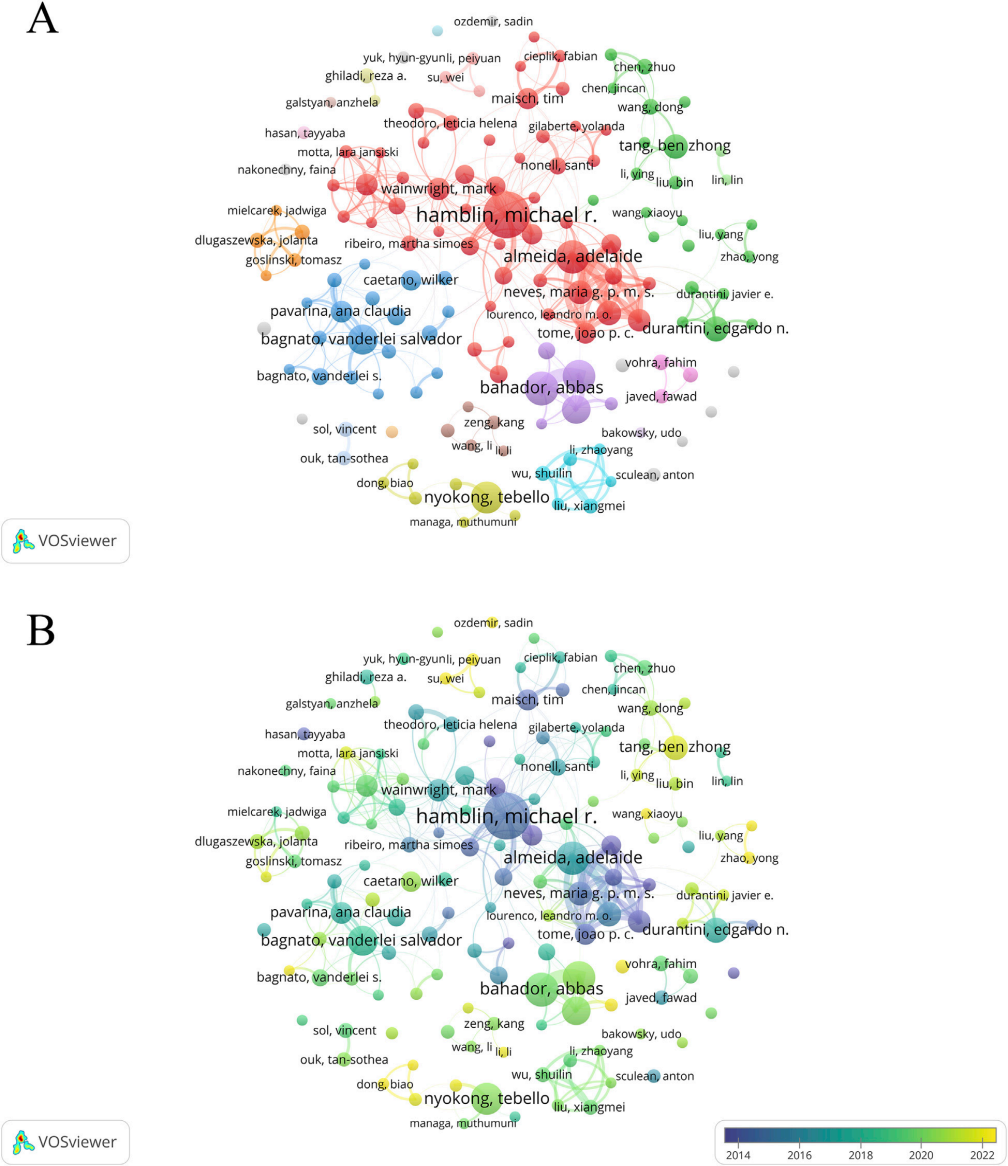
Analysis of authors. Analysis of authors. **(A)** Cooperation network diagram among authors with no fewer than 15 articles (n = 147); **(B)** the temporal map of collaboration network among authors with no less than 15 articles (n = 147).

**FIGURE 8:**
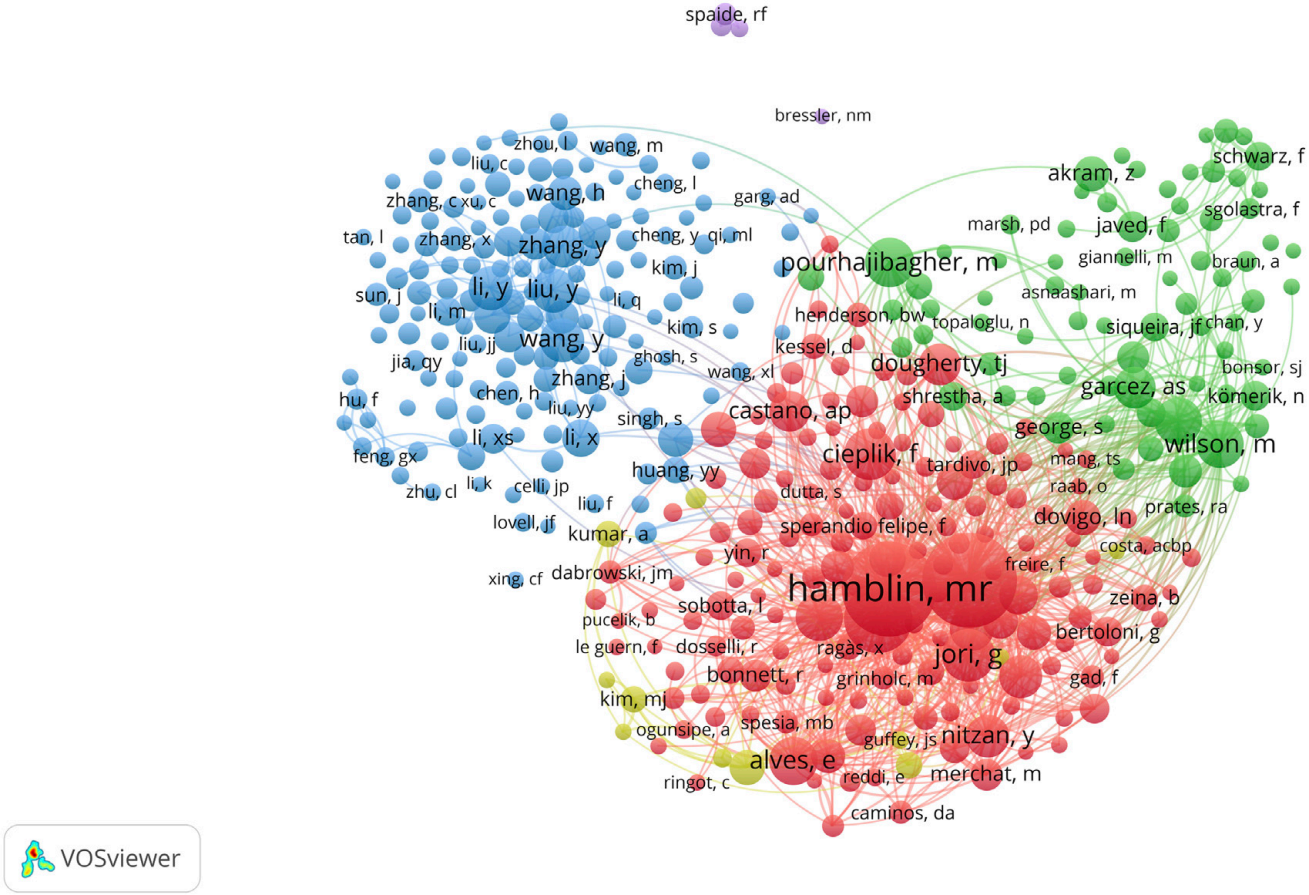
Analysis of authors. Co-citation analysis of authors with over 100 co-citations (n = 404).

A total of 40,081 authors have contributed to PDT research in antimicrobial therapy. Table 4 lists the top 10 most prolific authors. Michael R. Hamblin ranks first with 150 publications, leading in total citations (15,788), average citations per article (105.25), and H-index (77). The authors ranked second to 10th are Abbas Bahador (91), Adelaide Almeida (88), Maryam Pourhajibagher (85), Tebello Nyokong (82), Vanderlei S. Bagnato (74), Nasim Chiniforush (70), Edgardo N. Durantini (58), Maria Amparo F. Faustino (55), and Ben Zhong Tang (55). Although Tang and Faustino have relatively fewer publications, their high average citations and H-index (both top 5) highlight the impact and depth of their scholarly contributions. The analysis of the author collaboration network (Figures 7A,B) reveals that 147 scholars have collectively published over 15 relevant papers. Among these scholars, the group centered around Hamblin played a dominant role in pioneering early research in this field. In contrast, research activity among Chinese scholars has only seen a significant increase in recent years. Further analysis of the co-citation network (Figure 8) revealed that 404 authors were grouped into five distinct academic clusters. Hamblin (2,288 citations), Mark Wainwright, and Tim Maisch dominate in both co-citation frequency and Total Link Strength (TLS), constituting the structural core of the field’s citation network. In addition, we identified the ten most important articles in PDT-based anti-infective therapy (Table 5), further demonstrating the key literature that has shaped this research area.

**TABLE 4:** The top 10 authors based on the number of publications.

Rank	Authors	Publications	Percentage (n = 9711)	Total citations	Mean citations	H-index	Total link strength
1	Hamblin, MR.	150	1.54%	15788	105.25	77	220
2	Bahador, A	91	0.94%	1884	20.70	27	131
3	Almeida, A	88	0.91%	4742	53.89	40	315
4	Pourhajibagher, M	85	0.88%	1674	19.69	26	122
5	Nyokong, T	82	0.84%	1165	14.21	20	34
6	Bagnato, VS	74	0.76%	2176	29.41	33	100
7	Chiniforush, N	70	0.72%	1364	19.49	23	87
8	Durantini, EN.	58	0.60%	1788	30.83	29	64
9	Faustino, MAF.	55	0.57%	2657	48.31	41	245
10	Tang, BZ	55	0.57%	3653	66.42	31	41

**TABLE 5:** The top 10 most influential references in the application of PDT for infection control research.

Rank	Title	Authors	Journal	Citations	Year
1	Photodynamic therapy: a new antimicrobial approach to infectious disease?	Hamblin, MR	*Photochemical and Photobiological Sciences*	945	2004
2	Photodynamic antimicrobial chemotherapy (PACT)	Wainwright, M	*Journal of Antimicrobial Chemotherapy*	639	1998
3	Photodynamic therapy in the treatment of microbial infections: Basic principles and perspective applications	Jori, G	*Lasers in Surgery and Medicine*	484	2006
4	Antimicrobial photodynamic inactivation: a bright new technique to kill resistant microbes	Hamblin, MR	*Current opinion in microbiology*	399	2016
5	Photodynamic therapy for cancer	Dolmans, DEJGJ	*Nature Reviews Cancer*	398	2003
6	Photodynamic therapy for localized infections—State of the art	DAI, TH	*Photodiagnosis and photodynamic therapy*	390	2009
7	Antimicrobial photodynamic therapy–what we know and what we don't	Cieplik, F	*Critical reviews in microbiology*	376	2018
8	Photodynamic therapy of cancer: an update	Agostinis, P	*CA: a cancer journal for clinicians*	370	2011
9	Photoantimicrobials—are we afraid of the light?	Wainwright, M	*The Lancet Infectious Diseases*	367	2017
10	Photodynamic therapy in dentistry	Konopka, K	*Journal of dental research*	347	2007

### 3.6 Analysis of keywords

**FIGURE 9:**
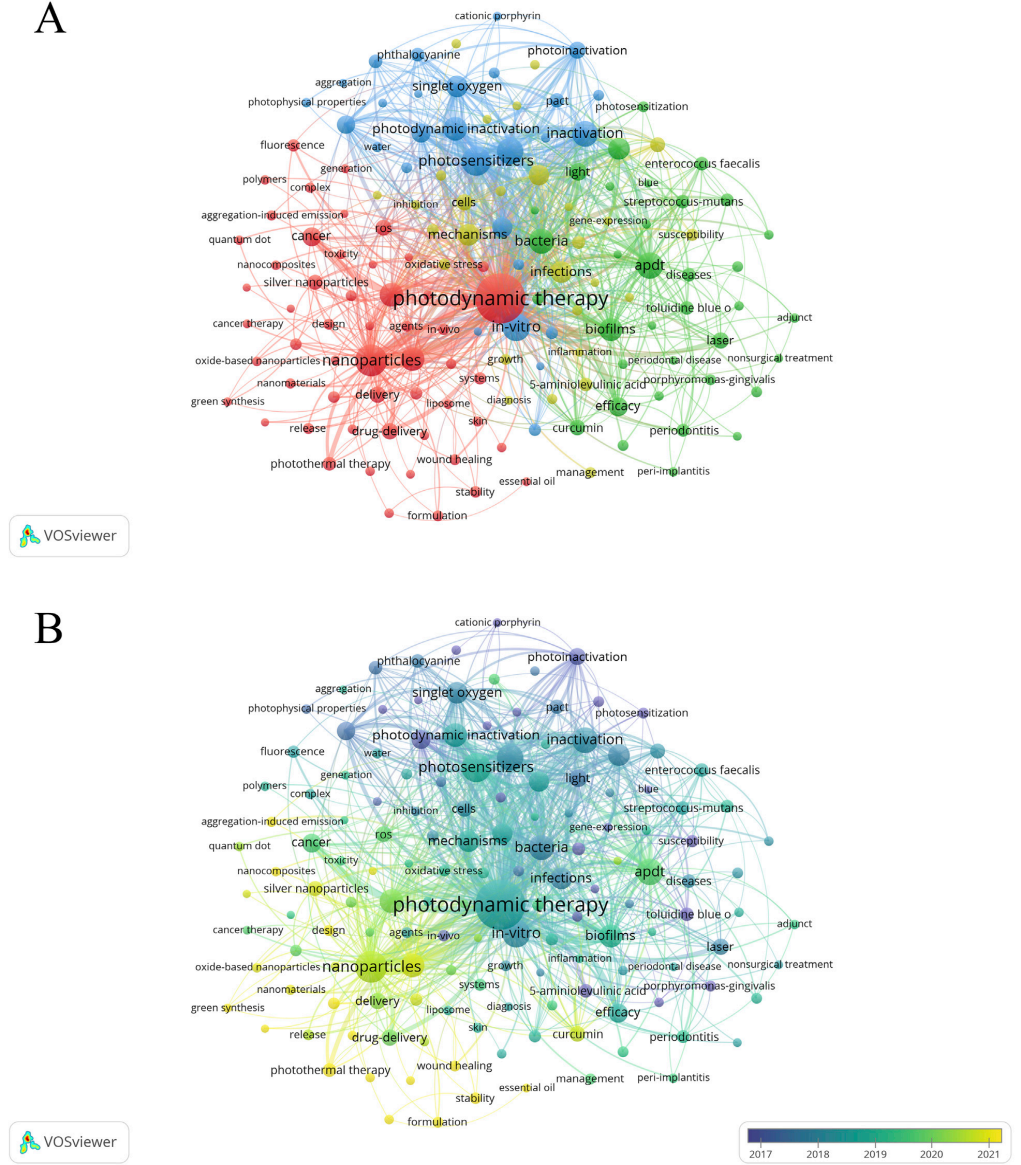
Analysis of keywords. **(A)** Co-occurrence network diagram of keywords with a frequency of no less than 90 occurrences (n = 146); **(B)** The overlay visualization map (n = 146).

**FIGURE 10:**
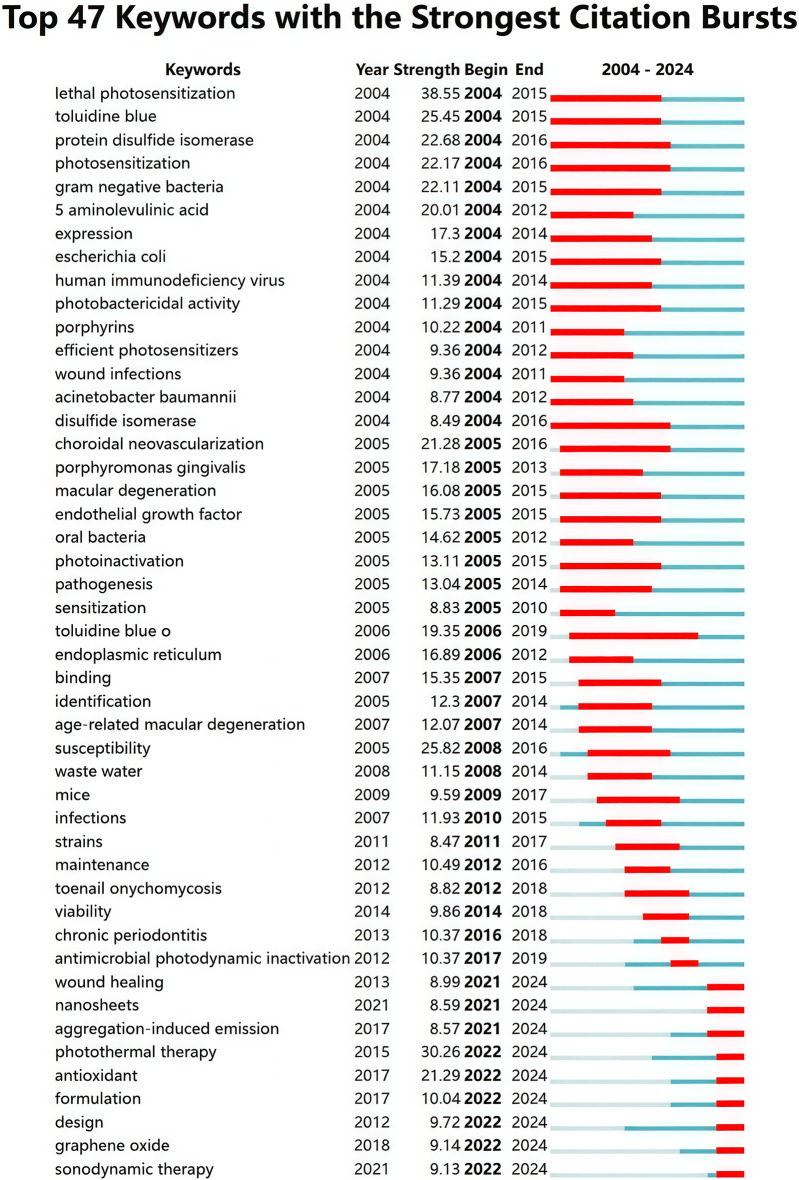
The top 47 keywords with the strongest citation bursts by CiteSpace.

After deleting meaningless words, merging synonyms and setting the minimum frequency of keywords to 90 times, a total of 146 keywords were obtained and then visualized using VOSviewer. Figure 9A shows that the target keywords are divided into four clusters (red, blue, green, and yellow). The top five keywords with the highest occurrence frequencies are “photodynamic therapy” (4,148), “nanoparticles” (1,611), “photosensitizers” (1,304), “*in vitro*” (1,222), and “therapy” (1,107). Given the time-sensitive nature of the research topic, a temporal overlay analysis of the keywords was conducted. As shown in Figure 9B, clusters in purple or blue represent early research themes in the field, while yellow clusters indicate current research hotspots. Common keywords in the early stages included “5-aminolevulinic acid,” “photoinactivation,” “porphyrins,” and “photosensitization.” With the continuous development of the field, terms such as “nanoparticle,” “antibacterial,” “photothermal therapy,” “nanomaterial,” “nanocomposite,” “aggregation-induced emission,” and “metal-organic framework” have emerged as new focal points of research. Furthermore, we employed CiteSpace to perform burst detection analysis of the keywords. As illustrated in Figure 10, a total of 47 keywords with the highest citation frequencies were identified. Among them, the two with the strongest burst intensities were “lethal photosensitization” (burst strength = 38.55) and “photothermal therapy” (burst strength = 30.26). Since 2021/2022, nine keywords—including “aggregation-induced emission,” “wound healing,” “photothermal therapy,” “sonodynamic therapy,” “antioxidant,” “formulation,” “design,” “nanosheets,” and “graphene oxide”—have shown continuous burst trends and have had a significant impact on the future research of PDT in the field of anti-infective treatment.

## 4 Discussion

In this study, we conducted a bibliometric analysis of 9,711 publications related to PDT for anti-infective treatment using VOSviewer and CiteSpace software. We comprehensively reviewed the current research status and hotspots within this field and predicted future research trends. Our results indicate a significant global increase in publication volume from January 2004 to December 2023, which can be divided into three phases: a period of steady growth (2004–2013), an accelerated phase (2014–2020), and a peak phase (2021–2023). The earliest foundational work on antimicrobial PDT was established by Raab in 1900, marking the inception of a century-plus of sustained scientific exploration (Raab, 1900). However, early research progress was relatively slow due to the inherent limitations of traditional PSs. The phenomenon of aggregation-induced emission (AIE), discovered in 2001, effectively addressed the challenge of PS aggregation and opened new avenues for the development of novel PSs (Luo et al., 2001; Abrahamse et al., 2022). Independent applications of nanotechnology, along with its integration with combination therapies, have driven significant advancements in the field (Youf et al., 2021; Yan et al., 2024). Since 2021, annual publication outputs have consistently surpassed 1,000 articles, with 1,308 publications in 2023—marking an increase of more than 17 times relative to the output in 2004. Notably, publication output from January to April 2024 has already exceeded the cumulative total for the same period over the past 5 years, indicating that the field remains in a phase of rapid growth, with increasing scholarly attention and the potential for sustained expansion in the foreseeable future.

As illustrated in Table 1, China dominates PDT research for anti-infective applications among the 126 analyzed countries/regions. It leads globally in publication volume, total citations, and H-index, underscoring its robust research capacity and academic influence in this field, followed by the United States and Brazil. In terms of average citations per article, the United States ranks first, reflecting the high quality and international impact of its research. China ranks fourth in this metric, suggesting that with ongoing improvements in its research environment, the country is steadily advancing toward higher-quality scientific output. In terms of international collaboration, China has also demonstrated outstanding performance, emerging as a key collaborative hub in the field alongside the United States and Brazil. The top 10 institutions by global publication output are distributed across the Americas, Asia, and Europe. The University of São Paulo and the Chinese Academy of Sciences rank first and second in publication volume, with corresponding global H-index rankings of fourth and third, respectively. Interestingly, although Massachusetts General Hospital and Harvard University have published fewer articles, they rank fourth and third, respectively, in total citations, average citations per article, and H-index—highlighting their substantial academic influence in this research domain. The institutional collaboration network depicted in Figure 3C reveals that several institutions from China, the United States, Brazil, and Iran are actively engaged in scholarly publishing, forming a significant research force in this field. However, their collaborations remain largely confined within national boundaries, with limited international interaction. This cooperation pattern may hinder cross-disciplinary communication and the development of innovation to some extent. Moving forward, fostering transnational collaboration, promoting knowledge sharing, and enhancing technological exchange will be essential to achieving higher-quality scientific research outcomes.

Journal analysis constitutes a critical component of bibliometric research, as significant studies in this field are often published in leading core journals. Accordingly, this study analyzed the most influential journals within the field. As presented in Table 3, the top 10 most productive journals collectively published 2,241 articles, representing 23.08% of total publications. The five highest-output journals were *Photodiagnosis and Photodynamic Therapy*, *Journal of Photochemistry and Photobiology B: Biology*, *Lasers in Medical Science*, *Photochemical and Photobiological Sciences*, and *Photochemistry and Photobiology*. Among them, *Photodiagnosis and Photodynamic Therapy* significantly outperformed the others in terms of publication volume, total citations, and H-index, underscoring its exceptional academic standing. *Journal of Photochemistry and Photobiology B: Biology* ranked second in publication count and shared the highest H-index with *Photodiagnosis and Photodynamic Therapy*. *Photochemical and Photobiological Sciences* stood out in terms of average citations per article, reflecting its strong research impact. When assessing journal influence through established metrics, the Journal IF and JCR classifications reveal significant insights. Per JCR quartile rankings based on IF, 50% of top 10 journals reside in Q1, with *ACS Applied Materials and Interfaces* possessing the highest IF (8.5). Notably, despite substantial research contributions from China and Brazil, the top 10 high-output journals lack representation from Asian or South American publishers. This observation underscores the necessity for enhanced development of internationally competitive academic platforms in these regions. Subject classification analysis indicates that the top 10 journals predominantly focus on the following disciplines: Biochemistry and Molecular Biology, Biophysics, Biomedical Sciences, Physical Chemistry, Materials Science, and Nanoscience and Nanotechnology, reflecting the primary research directions and thematic priorities in this field. Subsequent co-citation analysis reveals a substantial overlap between the five most frequently cited journals and the most productive ones. These journals include *Photodiagnosis and Photodynamic Therapy*, *Journal of Photochemistry and Photobiology B: Biology*, *Photochemistry and Photobiology*, *ACS Applied Materials and Interfaces*, and *ACS Nano*, further substantiating their scholarly authority and disciplinary influence. Looking forward, the core research outputs in this field are likely to continue appearing in these journals and will remain pivotal in shaping academic directions and advancing scientific progress.

Identifying prolific authors and their academic influence is essential for understanding the core forces and knowledge structure within a research field. Among the top 10 authors ranked by publication output, Hamblin stands out as the only scholar in this field with over 150 published papers, more than 15,000 total citations, and an average of over 100 citations per paper, securing the highest global H-index. His research primarily focuses on the mechanisms and applications of antimicrobial photodynamic inactivation (PDI) and aPDT (Hamblin and Hasan, 2004; Hamblin, 2016; Hamblin and Abrahamse, 2020). His seminal 2004 paper, titled “Photodynamic Therapy: A New Antimicrobial Approach to Infectious Disease?” (Hamblin and Hasan, 2004), remains highly cited. This work not only established the foundational theoretical framework of PDT in the field of anti-infection but also directly propelled the development and clinical translation of pathogen-specific PSs, such as cationic-modified compounds. Furthermore, he demonstrated that the antibacterial efficacy of PDT could be significantly enhanced by integrating nanoparticles or inorganic salts (Hamblin, 2017; Alipoor et al., 2022; Wen et al., 2017). Hamblin, widely regarded as the “father of modern phototherapy,” holds a prestigious international reputation for his pioneering contributions to the field. Amid the COVID-19 pandemic, Faustino and her colleagues proposed that using safe and cost-effective PSs, such as methylene blue (MB) and porphyrins, for aPDT may help alleviate COVID-19 symptoms (Almeida et al., 2020). Hamblin further suggested combining photobiomodulation therapy (PBMT) with lung-targeted nano- PSs, offering new ideas for combating COVID-19 and reducing dependence on antiviral drugs (Fekrazad et al., 2021). Scholars such as Almeida, Wainwright, and Maisch have critically examined the limitations of conventional antimicrobial strategies, highlighting the advantages of aPDT, including its multi-targeted attack mechanisms, broad-spectrum efficacy, and low propensity for inducing resistance, advocating for increased attention to this modality within the medical community (Wainwright et al., 2017). This academic initiative has garnered cross-regional support. Researchers from Tehran University of Medical Sciences—Bahador, Pourhajibagher, and Chiniforush—have been actively engaged in developing novel nanoparticles and nanocarrier systems with high biocompatibility, aiming to enhance the therapeutic efficacy of aPDT in pathogen eradication and biofilm disruption. Their work has significantly advanced the translational potential of this technology in the treatment of oral infections and refractory lesions (Gholibegloo et al., 2018; Akbari et al., 2017; Pourhajibagher et al., 2020). In addition, Bahador and Pourhajibagher have also focused on elucidating the mechanisms and improving the efficacy of antimicrobial sonodynamic therapy (aSDT), particularly enhancing its antibiofilm effects through nanotechnological approaches (Pourhajibagher and Bahador, 2021; Pourhajibagher et al., 2023; Bahrami et al., 2023). The innovative contributions from the Asia-Pacific region are equally remarkable. Professor Tang Benzhong from China, a pioneer in the field of AIE, and his team have systematically constructed a series of novel AIE PSs with type I ROS generation capabilities, high targeting ability, and good biocompatibility through molecular engineering strategies. These PSs effectively enhance the antibacterial efficacy against various pathogens, including MDR bacteria, Gram-negative bacteria, and *Mycobacterium tuberculosis* (Gong et al., 2023; Yu et al., 2023; Xiao et al., 2022). This work provides a representative “Chinese solution” for aPDT. The co-citation network analysis of the authors reveals that Wainwright, Hamblin, Maisch, and Altair S. de Almeida Alves, among others, form a tightly knit collaborative cluster. Their extensive contributions to high-impact journals and close collaborations have significantly advanced the application and development of PDT for infection control. Looking forward, they are expected to remain at the forefront of research in this field.

Keywords, as core elements of academic papers, not only help explore the current state of research in the target field but also predict research hotspots and development trends. To map the intellectual structure, a visual analysis of keywords was conducted using VOSviewer, revealing central themes in the global PDT anti-infective field. As shown in Figure 7B, early research in this domain primarily focused on the mechanisms of PSs, particularly their photochemical reactions during microbial inactivation (Wainwright, 1998). The emphasis was placed on evaluating the photochemical properties, toxicity, and safety of traditional PSs, such as porphyrins, phthalocyanines, and chlorins, and on verifying their bactericidal effects against various pathogens (such as bacteria and fungi) (O’Riordan et al., 2005; Javed et al., 2014), thus laying the foundation for the initial application of PDT in anti-infective treatment. As research progressed, biofilm-associated infections emerged as a key focus. This transition can be attributed to the fact that biofilm-embedded pathogens exhibit reduced susceptibility to conventional antibiotics and enhanced resistance mechanisms—challenges that aPDT is uniquely equipped to address. Indeed, a growing body of *in vitro* and *in vivo* evidence confirms that aPDT can effectively eradicate or substantially reduce microbial biofilms (Wang et al., 2022; Shi et al., 2021; Ribeiro et al., 2022; He et al., 2022). Despite the considerable advantages of PDT in anti-infective treatment, its practical application remains hindered by the limitations of traditional PSs, including poor target specificity, short-lived ROS, strict dependence on specific light conditions, and limited efficacy against Gram-negative bacteria (Polat and Kang, 2021).

To overcome the aforementioned limitations, researchers have significantly enhanced the antibacterial efficacy of aPDT through multidimensional strategies, including structural modification of PSs, development of novel PSs, and synergistic integration of enhancement techniques. Studies have demonstrated that cationic derivatives of phenothiazine, phthalocyanine, and porphyrin obtained through structural modifications significantly enhance PDI efficacy against both Gram-positive and Gram-negative bacteria (Yin and Hamblin, 2015), and the development or synthesis of new/natural PSs can further improve their photochemical properties and targeting capabilities (Polat and Kang, 2021; Zhou et al., 2023). Additionally, electroporation and chemical enhancers such as calcium chloride (CaCl_2_) and ethylenediaminetetraacetic acid (EDTA) can promote the uptake and accumulation of PSs by target bacteria, thereby amplifying the photodynamic bactericidal effect (de Melo Wde et al., 2013; Winter et al., 2013; Li et al., 2019). As a multifunctional platform for aPDT, nanomaterials overcome biological barriers by leveraging their tunable optical properties and versatile designs. Engineered as PSs, these optimized nanomaterials exert targeted antibacterial and anti-biofilm activity through multimodal mechanisms, including physical membrane disruption, oxidative stress via ROS generation, and intrinsic photodynamic effects. Representative materials include noble metal nanoparticles (e.g., Ag/Au NPs) (Liu et al., 2020; Xie et al., 2020; Huo et al., 2016; Mammari and Duval, 2023), metal oxides (e.g., ZnO, TiO_2_) (Siddiqi et al., 2018; Ziental et al., 2020), semiconductor quantum dots (McCollum et al., 2021), and carbon-based architectures such as graphene oxide (GO) and carbon dots (C-dots) (Nie et al., 2020; Perreault et al., 2015). The combined action of these functions shows their ability to fight drug-resistant bacteria and stubborn biofilm infections. Furthermore, innovations in drug delivery systems have propelled the advancement of aPDT. Carriers based on chitosan (Boroumand et al., 2021; Sinani et al., 2024), upconversion nanoparticles (UCNPs) (Hamblin, 2018), and liposomes (Zang et al., 2024) achieve lesion-targeted therapy through controlled PS release, maintaining antibacterial efficacy and biofilm clearance efficiency while minimizing dose-dependent toxicity (Gupta et al., 2019). Advancements in light source technology are equally crucial for optimizing the efficacy of aPDT. Effective aPDT implementation necessitates defined spectral overlap between the emission profile of the light source and the absorption spectrum of the PS to trigger phototoxic reactions. Laser and light-emitting diode (LED) technologies are widely employed to eradicate pathogens while minimizing damage to healthy tissues (Polat and Kang, 2021). Near-infrared (NIR) light, with its superior tissue penetration capability, provides improved therapeutic solutions for deep-seated infections when paired with compatible PSs (Vankayala and Hwang, 2018). Notably, nanotechnology not only optimizes the structural design of PSs but also enhances the overall therapeutic efficacy of aPDT by improving spectral absorption efficiency.

Building upon these developments, contemporary research has progressively shifted toward nanotechnology-based approaches, as evidenced by the clustering of keywords such as “nanoparticles,” “nanomaterials,” and “nanocomposites” in the time-series analysis (Figure 7B). These terms, along with emerging modalities including “photothermal therapy” and “aggregation-induced emission”, occupy prominent positions in the visualization, reflecting their status as current research priorities. Furthermore, a growing body of evidence has demonstrated that the combined use of PDT with conventional antibiotics (Pérez-Laguna et al., 2021; Branco et al., 2018), photothermal therapy (PTT) (Naskar and Kim, 2023; Cui et al., 2020), sonodynamic therapy (SDT) (Alves et al., 2021; Pang et al., 2020), and gas therapy (Fraix and Sortino, 2018; Zhu et al., 2021) enhances the overall therapeutic efficacy. The integration of nanotechnology further amplifies the complementary actions of these combinatorial therapies, maximizing antimicrobial efficacy and exhibiting pronounced activity against drug-resistant infections (Gao et al., 2023; Li et al., 2021; Hu et al., 2022). In conclusion, nanotechnology-based synergistic enhancement strategies for PDT and aPDT have become a dominant research focus in this field. However, challenges such as the complex design of nanosystems, high production costs, and unresolved issues related to targeting selectivity, biocompatibility, low toxicity, and long-term efficacy necessitate further investigation. Addressing these limitations will be critical for advancing the clinical translation of aPDT technologies.

Burst detection is a technique used to identify keywords that exhibit a significant increase in frequency over a specific period, often serving as an indicator for tracking dynamic changes at the forefront of a research field. During the study period, we identified the top 47 keywords with the highest burst strengths. Among them, “lethal photosensitization” showed the strongest citation burst, highlighting its central role and confirming that photosensitization-mediated pathogen elimination remains a key mechanism of aPDT (Hu et al., 2022). Notably, nine keywords, including “aggregation-induced emission,” “wound healing,” “photothermal therapy,” “sonodynamic therapy,” “antioxidant,” “formulation,” “design,” “nanosheets,” and “graphene oxide”, have been experiencing continuous bursts since 2021/2022. These topics likely represent the cutting edge of current research and may significantly influence future directions. In optical therapy, the success of aPDT heavily depends on the choice of PS. Traditional PSs such as porphyrins and phthalocyanines suffer from the aggregation-induced quenching (ACQ) effect, which diminishes ROS production. In contrast, PSs developed based on the phenomenon of AIE exhibit enhanced radiative transitions in the aggregated state, leading to increased ROS generation and improved therapeutic outcomes (Zhou et al., 2023). For instance, a Type I AIE PS developed by Professor Tang Benzhong's group achieved 99% photodynamic bactericidal efficiency against MRSA and *Staphylococcus aureus* (*S. aureus*) under low-dose light exposure, outperforming both conventional PDT and clinical antibiotics (Yu et al., 2023). Similarly, Guo et al. designed AIE nanofibers (AIE-NFs) that effectively eliminated pathogens both *in vitro* and *in vivo* while accelerating the healing of infected wounds (Guo et al., 2022). To address the high resistance of biofilm-associated infections, the introduction of two-dimensional (2D) nanomaterials including graphene derivatives, molybdenum disulfide (MoS_2_), Mxenes, and black phosphorus has added a new dimension to synergistic antimicrobial strategies (Xin et al., 2024). Using GO as an example, its ultrathin structure and programmable surface chemistry enable multiple mechanisms of action, including physical disruption (e.g., “nano-knife” membrane cutting), photothermal/photodynamic synergy (local heating combined with ROS bursts), and biofilm penetration, thereby significantly enhancing antimicrobial efficacy (Ng and Shamsi, 2022; Ramalingam et al., 2019). Experiments by Romero et al. showed that GO nanomaterials, under LED red light irradiation, could completely inactivate both Gram-positive and Gram-negative bacteria, while exhibiting significantly lower toxicity to human dermal fibroblasts (HDFs) compared to traditional PSs (Romero et al., 2020).

PTT and SDT are two other effective emerging antimicrobial strategies. SDT, derived from PDT, utilizes ultrasound (US) to activate sonosensitizers to produce ROS. Its main advantages lie in its deep tissue penetration and biofilm-disrupting capabilities, effectively overcoming the limitations of traditional PDT, which is generally restricted to superficial tissues (Xu et al., 2023; Li et al., 2021). The MCHH nanoclusters developed by Zhang’s group enhanced both biofilm permeability and local oxygenation, significantly improving SDT efficacy under US irradiation (Zhang et al., 2024). Similarly, PTT eradicates pathogens by locally increasing temperature to disrupt cell membranes and protein structures (Naskar and Kim, 2023). It is often combined with PDT to form dual-modal phototherapy. Recent studies have demonstrated that dual-modality phototherapy (combining PDT and PTT) not only reduces treatment duration and drug dosage but also synergistically enhances antibacterial efficacy (Huang et al., 2016; Huang et al., 2024). For example, Ru@MXene nanocomposites can achieve a local temperature increase to 50.4°C under light exposure, and when combined with ROS production, achieve near-100% bactericidal efficiency against *Escherichia coli* and *S. aureus* (Liu et al., 2023). It’s worth noting that the synergistic PTT/PDT approach not only exerts dual physical-chemical antimicrobial effects but also promotes wound healing by modulating immune responses, such as enhancing macrophage M2 polarization (Chen et al., 2023). Currently, PDT and its combination with antibiotics have demonstrated significant therapeutic efficacy in clinical treatments of brain abscesses and superficial infections such as cutaneous fungal infections and periodontal disease (Kharkwal et al., 2011; Gholami et al., 2023). In contrast, although nanomaterial-based multimodal synergistic antibacterial strategies have shown promising bactericidal potential in basic research, their clinical translation still faces multiple challenges. These include unresolved biosafety issues such as immunogenic responses to nanomaterials, poor stability in complex physiological environments and unpredictable metabolic clearance pathways, and potential organ accumulation toxicity (Tang et al., 2024; Holsapple et al., 2005; Xin et al., 2024). Additionally, the lack of standardized fabrication processes, unclear dose–response relationships, and the absence of unified evaluation systems further hinder their clinical application (Parvin et al., 2024; Rashed et al., 2021). Therefore, future research should focus on optimizing nanomaterial synthesis to improve biocompatibility, developing biodegradable PSs and targeted delivery systems, establishing dynamic visualization tools and standardized therapeutic evaluation frameworks, and promoting interdisciplinary collaboration to overcome the challenges of large-scale production and regulatory adaptation.

## 5 Limitations

This study presents several significant findings; however, the following limitations should be considered when interpreting the results. First, only the WoSCC database was used as the data source, which may have led to the omission of relevant literature from other databases, such as Scopus, PubMed, and Google Scholar, particularly studies published in non-English or lower-impact journals. Second, only original articles and review articles written in English were included, which may have excluded important publications in other types or languages. Nevertheless, WoSCC, with its extensive literature resources and recognized authority, is still widely considered the most suitable and recommended database for bibliometric analysis. Furthermore, given that citation accumulation takes time, recently published high-quality papers may not yet have received sufficient citations to be fully represented. The timeliness of bibliometric analysis also implies that research trends and hot spots may evolve as databases are updated. Therefore, the findings of this study reflect only the overall status of research on PDT for anti-infection from January 2004 to April 2024.

## 6 Conclusion

In summary, this study systematically analyzed the application status and global development trends of PDT in the field of anti-infection using bibliometric methods. Over the past 20 years, global research in this field has significantly increased, with China undoubtedly occupying a central position, ranking first in both the number of publications and total citations. The United States ranks second and maintains a leading influence. Cooperation between institutions is primarily domestic, while international collaboration is mainly concentrated among China, the United States, and Brazil. In the future, researchers should further strengthen collaboration among different countries and institutions. *Photodiagnosis and Photodynamic Therapy* is the highest-producing journal in this field, and many highly cited papers have also been published in this journal. Hamblin is recognized as one of the most influential scholars in this area. Through keyword analysis, we believe that nanotechnology-based synergistic enhancement strategies for PDT and aPDT represent an important current research hotspot. Meanwhile, topics such as sonodynamic therapy, aggregation-induced emission, photothermal therapy, design, nanosheets, and graphene oxide are likely to remain at the forefront of research in this field in the coming years, warranting significant attention.

## Data Availability

The original contributions presented in the study are included in the article/Supplementary Material, further inquiries can be directed to the corresponding author.
